# Analysis of* E. rutaecarpa* Alkaloids Constituents* In Vitro* and* In Vivo* by UPLC-Q-TOF-MS Combined with Diagnostic Fragment

**DOI:** 10.1155/2016/4218967

**Published:** 2016-06-30

**Authors:** Shenshen Yang, Meng Tian, Lei Yuan, Haoyue Deng, Lei Wang, Aizhu Li, Zhiguo Hou, Yubo Li, Yanjun Zhang

**Affiliations:** ^1^School of Traditional Chinese Materia Medica, Tianjin University of Traditional Chinese Medicine, 312 Anshan West Road, Tianjin 300193, China; ^2^Tianjin State Key Laboratory of Modern Chinese Medicine, Tianjin University of Traditional Chinese Medicine, 312 Anshan West Road, Tianjin 300193, China

## Abstract

*Evodia rutaecarpa* (Juss.) Benth. (Rutaceae) dried ripe fruit is used for dispelling colds, soothing liver, and analgesia. Pharmacological research has proved that alkaloids are the main active ingredients of* E. rutaecarpa*. This study aimed to rapidly classify and identify the alkaloids constituents of* E. rutaecarpa* by using UPLC-Q-TOF-MS coupled with diagnostic fragments. Furthermore, the effects of the material base of* E. rutaecarpa* bioactive ingredients* in vivo* were examined such that the transitional components in the blood of rats intragastrically given* E. rutaecarpa* were analyzed and identified. In this study, the type of alcohol extraction of* E. rutaecarpa* and the corresponding blood sample were used for the analysis by UPLC-Q-TOF-MS in positive ion mode. After reviewing much of the literature and collected information on the fragments, we obtained some diagnostic fragments of the alkaloids. Combining the diagnostic fragments with the technology of UPLC-Q-TOF-MS, we identified the compounds of* E. rutaecarpa* and blood samples and compared the ion fragment information with that of the alkaloids in* E. rutaecarpa*. A total of 17 alkaloids components and 6 blood components were identified. The proposed method was rapid, accurate, and sensitive. Therefore, this technique can reliably and practically analyze the chemical constituents in traditional Chinese medicine (TCM).

## 1. Introduction

*Evodia rutaecarpa* (Juss.) Benth. (Rutaceae) dried ripe fruit is used for dispelling colds, soothing liver, and analgesia [1].* E. rutaecarpa* can ease depression, relieve dyspepsia, and exert analgesic, sedative, antibacterial, and antioxidant activities, as well as other pharmacological effects. Up to now, alkaloids are the main active ingredients of* E. rutaecarpa* [2]. However, previous studies have focused on the indole biological components of* E. rutaecarpa* [3, 4]. TCM contains many components, but only the ingredients absorbed into the bloodstream produce an effect [5]. Therefore, the ingredients of TCM found in the animal blood should be analyzed using a vast majority of the drugs administered to animals to determine the active sites of actual and effective ingredients. The chemical constituents and absorbed components of* E. rutaecarpa* should be analyzed to completely control the medicinal qualities of this plant and simultaneously detect its main ingredients.

Previous studies have employed TLC, HPLC with UV detector or MS, and CE to quantify or identify the alkaloids in Evodia [6–10]. An effective and reliable analytical method should be established to quickly classify and identify* E. rutaecarpa* chemical compositions* in vitro* and* in vivo*. Ultraperformance liquid chromatography quadrupole time-of-flight mass spectrometry (UPLC-Q-TOF-MS) is advantageous because of its high speed, high resolution, and high accuracy. MS scanning can provide accurate mass information on characteristic molecular ions and fragment ions to offer a reliable basis for the qualitative and quantitative analyses of target compounds [11–13]. UPLC-Q-TOF-MS is also increasingly applied in many areas, which analyzes steryl glycosides and uncovers the effects of light intensity and temperature under shading treatments on the metabolites in tea [14, 15]. Diagnostic fragment was the characteristic fragment of a certain type of compound applied for screening and identification. Based on these characteristics, we also employed UPLC-Q-TOF-MS in our experiment to quickly classify and identify the alkaloids compositions of* E. rutaecarpa*. Additionally, we detected the absorbed components after intragastric administration by* E. rutaecarpa* alcohol extract and observed the exogenous active substances.

The above screening method can quickly classify and identify* E. rutaecarpa* chemical compositions. The proposed method provided a fast, accurate, and feasible analytical process to distribute and determine the complex components in traditional Chinese medicine (TCM). First, we used UPLC-Q-TOF-MS to perform a fingerprint analysis of* E. rutaecarpa* and obtain the fragments' information of* E. rutaecarpa* chemical constituents. Afterward, we rapidly classified and identified the* E. rutaecarpa* alkaloids compositions by using diagnostic fragments as screening tools. Finally, combined with the literature and standard we could confirm the compounds in the plant. According to this method, a total of 17 alkaloids constituents and 6 absorbed components were identified from* E. rutaecarpa*. The established method is expected to be widely accepted and approved, given the popularity of MS and the requirements of fast and efficient assays in routine studies.

## 2. Materials and Methods

### 2.1. Materials

The UPLC-Q-TOF-MS system (Waters, USA) consisted of an autosampler and a DAD detector and a column compartment was used for the analysis. Acetonitrile, formic acid, and methanol were chromatographically pure. The 95% alcohol used was also analytically pure and* E. rutaecarpa* (Juss.) Benth. adopted in this experiment was purchased from Hubei Herbs Company. A total of 10 male Wistar rats weighing 180 ± 20 g were obtained from the Academy of Military Medical Sciences.

### 2.2. UPLC-Q-TOF-MS Analysis Conditions

The chromatographic column used was Waters ACQUITY UPLC BEH C_18_ Column (2.1 × 100 mm, 1.7 *μ*m). Column temperature was set to 35°C. Phase A was the water phase, which consisted of 0.1% aqueous solution of formic acid. Phase B consisted of 0.1% formic acid in acetonitrile solution at a flow rate of 0.3 mL/min and injection volume of 5 *μ*L. Gradient elution was used for chromatographic separation and the gradient programs were 0-1 min, 11% B; 1-2 min, 11%–21% B; 2–4 min, 21%–33% B; 4–7 min, 33%–70% B; 7–9 min, 70%–82% B; 9–16 min, 82%–100% B; 16–18 min, 100% B; 18-19 min, 100%–11% B; and 19-20 min, 11% B.

UPLC-Q-TOF-MS equipped with ESI in positive ion mode was used to scan and analyze the chemical constituents of* E. rutaecarpa*. MS parameters were set as follows: dry gas (N_2_) flow rate, 10 mL/min; gas temperature, 325°C; desolvation gas flow rate, 600 L/h; capillary voltage, 2.1 kV; and collision-induced dissociation voltage, 6 kV. For the nebulizer, the boil-off gas was at 350 psi of pressure and the auxiliary gas was high-purity nitrogen. The reference ion was [M+H]^+^ = 556.2771 to ensure the acquisition accuracy in spectra. Data were acquired in the range of 50–1000 Da.

### 2.3. Solution Preparation

*E. rutaecarpa* powder (40 mesh) was extracted twice by refluxing extraction. The first extraction was performed 10 times with 70% ethanol extract for 1.5 h and the second extraction was 8 times of 70% ethanol extract for 1.5 h. After filtration, the mixed filtrate was concentrated to a relative density of 0.1 g crude drug/mL by recovering ethanol, and the mixture was stored for further use.

### 2.4. Blood Samples Preparation

Ten Wistar rats (180 ± 20 g) were fasted with free access to water for 12 h prior to the experiment. The rats were then intragastrically given* E. rutaecarpa* ethanol extract (0.6 g crude drug/kg body weight). After 2 h, blood samples were collected from the eye venous plexus. The samples were centrifuged at 3000 r/min for 15 min and 3500 r/min for 8 min to obtain the serum. The serum was immediately placed in a refrigerator at −80°C and stored until analysis. 200 *μ*L of the serum was precisely pipetted and placed in a 5 mL stoppered centrifuge tube. An equivalent amount of acetonitrile was added to the tube for protein precipitation. After stirring for 3 min, the tube was centrifuged at 4°C at 13000 r/min for 15 min. Supernatant was filtered through a 0.45 *μ*m membrane for UPLC-Q-TOF-MS analysis in accordance with chromatographic conditions.

## 3. Results and Discussion

### 3.1. Established Methods of Compound Classification and Identification

**Figure 1:**
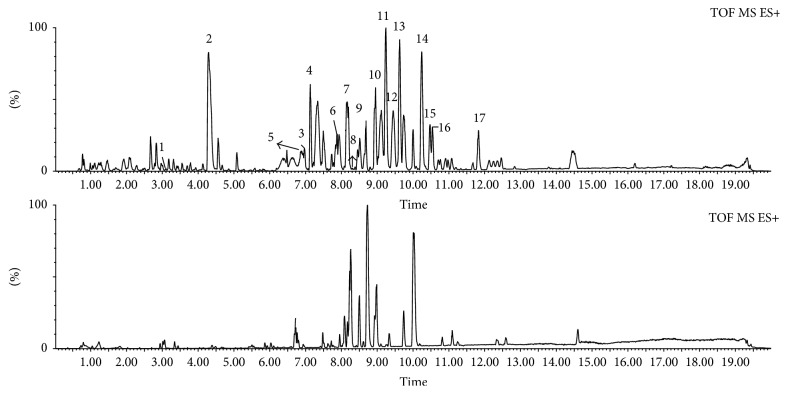
Typical total ion current (TIC) chromatograms of alkaloids constituents in* E. rutaecarpa* and serum under positive ion mode.

TCM involves many plants with complex chemical compositions. However, substances with similar frameworks are placed in the same category, and these substances exhibit similar material fracture behavior during collision-induced mass spectrometry. Therefore, we used this feature to explore for fragmentation patterns of a substance in its mass spectrum [16]. Based on the structural characteristics of the two alkaloids and the information collected from a large number of literatures, and compared with the standard of mass spectrometry, the rule of the diagnosis of these two kinds of alkaloids was found and summarized. Therefore we established the method which combined diagnose fragments with UPLC-Q-TOF-MS to characterize the chemical compositions of* E. rutaecarpa*. As previously mentioned, alkaloids are the main active ingredients of this herb. The alkaloids in* E. rutaecarpa* are mainly indole alkaloids and quinolone alkaloids.  Figure 1 shows the typical total ion current (TIC) chromatograms of the results obtained via UPLC-Q-TOF-MS of this herb and blood.

We quickly identified the composition of* E. rutaecarpa* and speculated the possible structures of its compounds through their mass spectral fragmentation patterns and by combining the data reported in the literature (Table 1). A total of 5 indole alkaloids and 12 quinolone alkaloids were identified on the basis of the contrasting cleavage rules, fragment ion characteristics, and mass spectral data. In the present study, experimental rats were given intragastrically* E. rutaecarpa*. Serum was analyzed via UPLC-Q-TOF-MS coupled with serum medicinal chemical analysis to identify the constituents absorbed into the blood, and 6 components were identified in the blood.

### 3.2. Identification of the Compounds in* E. rutaecarpa* Herb

#### 3.2.1. Indole Alkaloid

We divided these alkaloids into two categories based on their structural characteristics. Category A contained one indole quinoline alkaloid with five complete rings. Category B contained ring openings instead of five ring structures in their parent nucleus.

According to the literature and reference mass spectrum information of standard available, the significant structural feature of category A is the transformation of the cyclohexene. Under the bombardment of MS collision voltage, this structure is prone to RDA fragmentation and characteristic ion peak formation, which are important to evaluate alkaloid structures. If the left side structures of RDA cleavage sites did not change the functional groups, *m*/*z* 171 was the diagnostic fragment produced after RDA fragmentation. Zhou et al. [17] used LC-ESI-MS^*n*^ to analyze evodiamine and rutaecarpine, which were the main ingredients in* E. rutaecarpa*. Zhou et al. [18] also found that, in the mass spectrum where neutral loss appeared, fragment ions, [M+H-CH_4_]^+^, [M+H-NH]^+^, [M+H-CO]^+^, and [M+H-H_2_O]^+^, as well as those formed by RDA fragmentation, were produced, which provided detailed structural information to detect unknown alkaloids in* E. rutaecarpa* samples.

**Figure 2:**
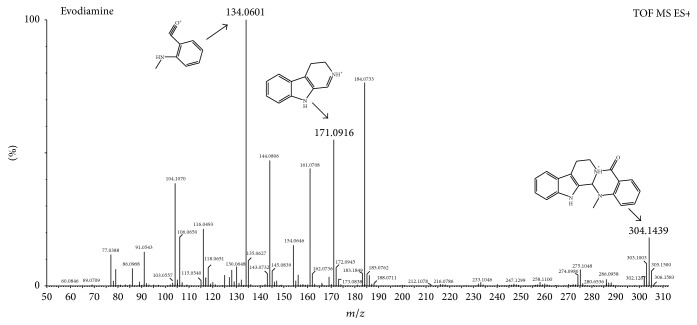
The mass spectrum of evodiamine in indole alkaloids.

The mass spectrum of compound 3 (Figure 2) showed the retention time at 6.97 min and estimated formula was C_19_H_17_N_3_O. The mass charge ratios of the main fragments were 304, 171, 161, 144, 134, 116, and 106; *m*/*z* 304 was the quasimolecular ion peak [M+H]^+^. We get the fragment ion at *m*/*z* 171 [M+H-C_8_H_8_NO]^+^ and *m*/*z* 134 [M+H-C_11_H_10_N_2_]^+^ ion molecule of complementary fragment ions which is the typical RDA fragmentation characteristics, so determining that it belongs to category A firstly. Furthermore, fragment ions at *m*/*z* 161 [M+H-C_10_H_10_N]^+^ and 144 [M+H-C_9_H_8_N_2_O]^+^ were found because another RDA fragmentation occurred, which resulted from the rearrangement of intramolecular double bonds. In addition, the *m*/*z* 144 [M+H-C_9_H_8_N_2_O]^+^ and 134 [M+H-C_11_H_10_N_2_]^+^ of debris were missing a -C_2_H_4_ and -CO formed *m*/*z* 116 [M+H-C_9_H_8_N_2_O-C_2_H_4_]^+^ and 106 [M+H-C_11_H_10_N_2_-CO]^+^ of the fragment ion. Hence, compound 3 was identified as evodiamine, which was consistent with the literature and standard [19]. At the same time, these alkaloids also contain rutaecarpine, 14-formyldihydroxyrutaecarpine, and dehydroevodiamine.

Category B showed significant structural differences from category A. RDA fragmentation did not occur in category B. These characteristics were used to distinguish the two categories. Consider that the cleavage active sites were amide and quaternary ammonium bonds. From the structures of category B, the easily broken sites were the C-N bonds in the amide groups. Other bonds linked to quaternary ammonium nitrogen atoms were also easy to break.

**Figure 3:**
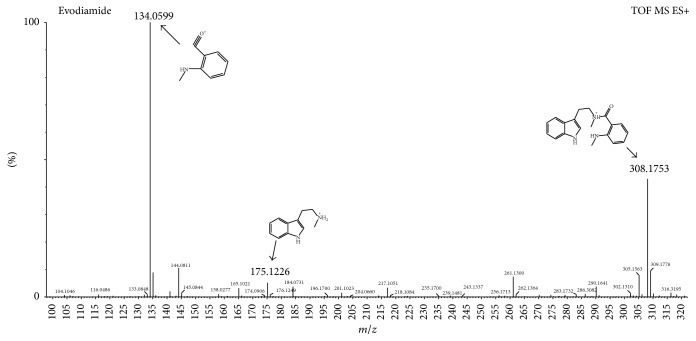
The mass spectrum of evodiamide in indole alkaloids.

The mass spectrum of compound 5 (Figure 3) showed the retention time at 6.81 min; estimated formula was C_19_H_21_N_3_O. The mass charge ratios of the main fragments were 308, 175, 165, 144, and 134, which is not the typical RDA fragmentation characteristics, so determining that it belongs to category B firstly. Among them, *m*/*z* 308 was the quasimolecular ion peak [M+H]^+^. *m*/*z* 134 [M+H-C_11_H_14_N_2_]^+^ and 175 [M+H-C_8_H_7_NO]^+^ were produced by amide structure fragmentation, which was the main fragmentation pathway of category B. Furthermore, fragment ions at *m*/*z* 165 [M+H-C_10_H_9_N]^+^ and 144 [M+H-C_9_H_12_N_2_O]^+^ were found resulting in the quaternary amine bond cleavage. Therefore, compound 5 was identified as evodiamide by comparing the mass spectral data with the related literature [20].

#### 3.2.2. Quinolone Alkaloids

The mother nuclei of quinolone alkaloids were identical, and they only differed in the position of C-2 in the side chains. According to the structure and literature known that quinolone alkaloids can occur replacement rearrangement and Mclafferty rearrangement lose part of side chain formation with a stable total conjugate system of fragment ions *m*/*z* 186 and *m*/*z* 173. Therefore, *m*/*z* 186 and 173 ion peaks were used as diagnostic fragments to evaluate quinolone alkaloids. Except for their identical mother nuclei, these compounds only differ in the position of the C-2 of side chains, where their carbon chain lengths and unsaturation degrees are distinct. However, only the fragment ions of carbon chain were difficult to detect, and the characteristic fragment ions of certain compounds were consequently absent in the mass spectral detection of quinolone alkaloids [21]. Thus, we documented the molecular ion peak [M+H]^+^ for authentication and identified quinolone alkaloid chemical constituents combined with information from the literature and mass spectral data. Hence, we preliminarily determined 12 quinolone alkaloids.

**Figure 4:**
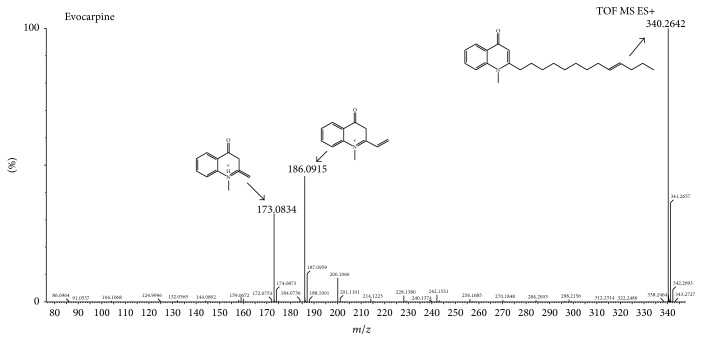
The mass spectrum of evocarpine in quinolone alkaloids.

Compound 11, for example, with a retention time at 9.24 min and inference formula, may be C_23_H_33_NO. In the experiment get *m*/*z* 340, 328, 187, 186, 174, 173, 159, 144, and 132 fragment ions. Firstly, the mother nuclear fragment ions with high abundance at *m*/*z* 186 [C_12_H_12_NO]^+^ and 173 [C_11_H_11_NO]^+^ were found to be unrelated to the side chains, which can be diagnostic compounds 11 belonging to quinolone alkaloids. Secondly, we also received by the *m*/*z* 144 and 159 of debris. The mass difference between *m*/*z* 186 [C_12_H_12_NO]^+^ and the fragment at *m*/*z* 144 [C_12_H_12_NO-C_2_H_2_O]^+^ indicated a lost ketene molecule. A fragment ion at *m*/*z* 159 [C_11_H_11_NO-CH_2_]^+^ was formed because a molecule of -CH_2_ was removed from the fragment at *m*/*z* 173 [C_11_H_11_NO]^+^. In addition, the mass spectrum of compound 11 showed a quasimolecular ion peak at *m*/*z* 340 [M+H]^+^; therefore, compound 11 was identified as evocarpine by comparing the mass spectral data with the standard. The major fragment ions of evocarpine are shown in Figure 4.

From the mass spectra of compounds 6–17, the mother nuclear fragment ions with high abundance at *m*/*z* 186 and 173 were found to be unrelated to the side chains. Thus, compounds 6–17 were quinolone alkaloids.

### 3.3. Identification of the Compounds in Blood

In this study, 6 alkaloids were identified in the blood. The 6 active components were dehydroevodiamine, evodiamine, rutaecarpine, and 14-formyldihydroxyrutaecarpine of indole alkaloids and evocarpine and dihydroevocarpine of the quinolone alkaloids. Through consulting the literature known, evodiamine and rutaecarpine in human serum via LC*–*MS by analyzing the blood components in* E. rutaecarpa* [22].

**Figure 5:**
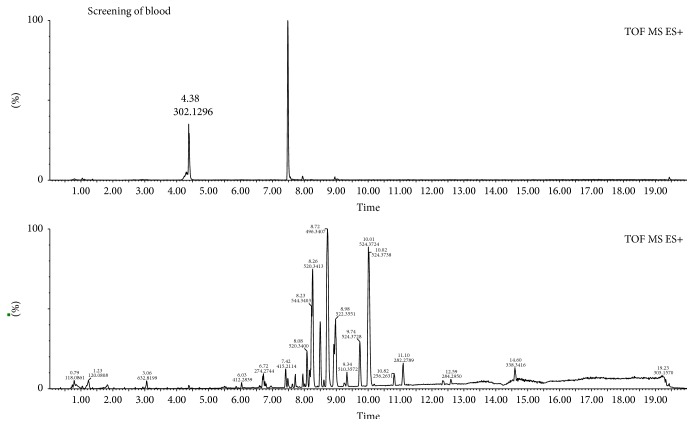
The screening result of blood sample.

**Figure 6:**
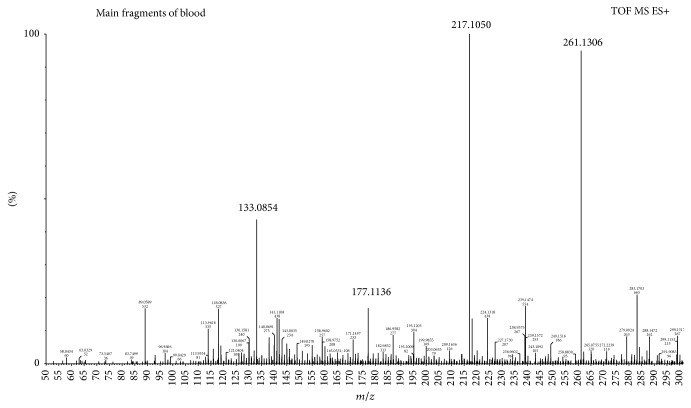
The main fragments of screening from blood.

**Figure 7:**
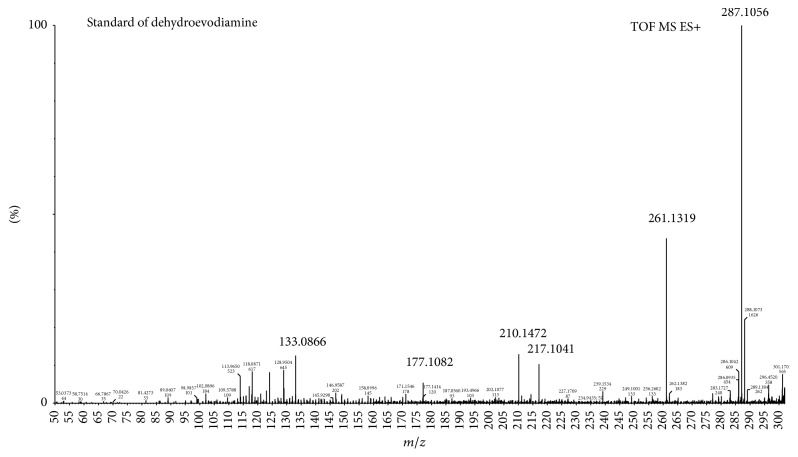
The main fragments of standard of dehydroevodiamine.

*Compound 2 as an Example.* Compound 2, a known compound in* E. rutaecarpa*, is dehydroevodiamine; the retention time is at 4.29 min, and estimated formula was C_19_H_15_N_3_O and the *m*/*z* of [M+H]^+^ is 302.1309. Firstly, screening for the data of blood samples using MassLynx software, the results of screening are that the *m*/*z* of [M+H]^+^ is 302.1296 and retention time is at 4.38 min; based on the retention time and the quasimolecular ion peak we can initially determine which is dehydroevodiamine. The screening result was shown in Figure 5. Then, the main fragments of screening compound were compared with the fragments of compound 2. The mass charge ratios of the main fragments of compound 2 were 302, 286, 261, 217, 177, and 133, and the mass charge ratios of the main fragments in screen compound were 302, 261, 217, 177, and 133. The debris information is basically consistent with the fragment information of compound 2. Finally, comparing the main fragments of screen compound with the standard, the mass charge ratios of the main fragments of dehydroevodiamine were 302, 287, 261, 217, 210, 177, and 133, which were consistent with the compound in blood samples, so determined the compound that the *m*/*z* of [M+H]^+^ is 302.1296 of screening in blood samples was dehydroevodiamine. The main fragments were shown in Figures 6 and 7.

Combining the diagnostic fragments with the technology of UPLC-Q-TOF-MS and comparing the ion fragment information with that of the alkaloids in* E. rutaecarpa*, a total of 17 chemical components were identified. At the same time, the data of blood samples by using UPLC-Q-TOF-MS were compared with the identified chemical components in* E. rutaecarpa* and we classified and identified the 6 active components of* E. rutaecarpa*. Thus, this method can quickly and accurately identify the main components and active components of the traditional TCM.

### 3.4. Discussion

In this experiment, 17 compounds were identified in* E. rutaecarpa*. After animals were intragastrically given* E. rutaecarpa*, 6 absorbed components were found. The existence of other forms of metabolites remains to be further verified.

We investigated* E. rutaecarpa* constituents and blood components in positive and negative ionization modes. After we detected the alkaloids in* E. rutaecarpa*, we observed a high abundance of [M+H]^+^ ion peaks in the excimer (+) ESI mass spectra, whereas [M−H]^−^ signals nearly did not appear in the excimer (−) ESI mass spectra. Thus, mass spectrum in positive ion mode was finally obtained, which was consistent with the literature [23].

There are different methods established for analysis of the chemical composition of Evodia reported in previous studies. One is use of HPLC-ESI-MS to identify five kinds of alkaloid in Evodia [24]; another is use of ESI-IT-TOF-MS method to analyze rutaecarpine and fragmentation pathway of the two derivatives based on accurate ion mass and multilevel spectroscopy [25]. Compared with the above two documents, this study not only combined the advantages of their method, using UPLC-Q-TOF-MS combined with fragments diagnostic technology, through the diagnostic fragments for rapid compound classification, but also can more accurately identify the chemical composition sensitively, and our research identified 17 chemical composition of Evodia.

In this study, the 6 active components were dehydroevodiamine, evodiamine, rutaecarpine and 14-formyldihydroxyrutaecarpine of indole alkaloids and evocarpine and dihydroevocarpine of the quinolone alkaloids. Evodia has analgesic, anti-inflammatory, antiulcer, relaxing blood vessels and lowering blood pressure, cardiac, antitumor, and other pharmacological effects. According to the literature, evodiamine from* Evodia rutaecarpa* induces apoptosis via activation of JNK and PERK in human ovarian cancer cells [26]. Evodiamine selectively targets cancer stem-like cells through the p53-p21-Rb pathway and may be used for the treatment of breast cancer [27]. Dehydroevodiamine could antagonize triggered arrhythmias such as ACs and DADs induced by cardiotonic agents in human atrial and ventricular tissues through a general reduction of the Na^+^ and Ca^2+^ inward currents, with an increase of pH_*i*_ and NHE activity [28]. Therefore, the role of these active ingredients is consistent with pharmacological effects of Evodia.

The mass spectral fragment ions were produced by the single-molecule cleavage reaction of molecular ions or large fragment ions. The abundance of a specific fragment ion relative to the molecular ions and other fragment ions provided the position structures in the molecules, environment, and other valuable information on fragment ions. UPLC-Q-TOF-MS is characterized by high analysis speed, high examination sensitivity, and strong anti-interference ability, among others. This process can effectively reduce body interference and improve detection accuracy when analyzing complex samples. This process is also applied to the simultaneous determination of various complex components in TCM. Furthermore, UPLC-Q-TOF-MS is extensively used to determine the structures of compounds. This method can quickly classify compounds and plays important roles in the identification of effective materials and components of TCM.

## 4. Conclusion

A rapid and sensitive method was developed using UPLC-Q-TOF-MS coupled with diagnostic fragment technique to separate and identify* E. rutaecarpa* chemical constituents* in vitro* and absorbed components* in vivo*. A total of 17 chemical constituents* in vitro* and 6 absorbed components* in vivo* were successfully identified in* E. rutaecarpa* in terms of the precise relative molecular mass, mass spectral fragment structural information, chromatographic retention rules of the chromatographic peaks in the mass spectra, and the previous literature. UPLC-Q-TOF-MS rapidly analyzed the chemical constituents and medicinal components of TCM and provided a new concept for the in-depth study on the medicinal composition of TCM. Hence, the proposed method is a relatively good qualitative determination technique to establish an experimental basis for further analysis of* E. rutaecarpa* chemical constituents, control of medicinal qualities, and development of TCM chemical constituents. Furthermore, this research on absorbed components* in vivo* is important for clinical research.

## Figures and Tables

**Table 1:** Characterization of chemical constituents of Evodia by UPLC-Q-TOF-MS.

Number	RT	Identification	Formula	Theoretical mass (*m*/*z*)	Experimental mass (*m*/*z*)	Error (ppm)	Blood components	MS data(+ ) (*m*/*z*)
1	3.07	14-Formyldihydroxyrutaecarpine	C_19_H_15_N_3_O_2_	318.1243	318.1245	0.63	Y	273, 261, 217, 171, 144
2	4.29	Dehydroevodiamine	C_19_H_15_N_3_O	302.1295	302.1309	4.63	Y	302, 286, 261, 217, 177, 133
3	6.97	Evodiamine	C_19_H_17_N_3_O	304.1450	304.1439	−3.62	Y	304, 171, 161, 144, 134, 116, 106
4	7.13	Rutaecarpine	C_18_H_13_N_3_O	288.1137	288.1141	1.39	Y	288, 273, 171, 169, 120
5	6.81	Evodiamide	C_19_H_21_N_3_O	308.1763	308.1753	−3.24	N	308, 175, 165, 144, 134
6	7.87	1-Methyl-2-nonyl-4(1H)-quinolone	C_19_H_27_NO	286.2171	286.2171	0	N	286, 261, 217, 186, 173, 144, 102
7	8.20	1-Methyl-2-[(Z)-6-undecenyl]-4(1H)-quinolone/1-methyl-2-[(Z)-5-undecenyl]-4(1H)-quinolone	C_21_H_29_NO	312.2327	312.2334	2.24	N	312, 217, 186, 173, 144
8	8.31	1-Methyl-2-[(Z)-6-undecenyl]-4(1H)-quinolone/1-methyl-2-[(Z)-5-undecenyl]-4(1H)-quinolone	C_21_H_29_NO	312.2327	312.2321	−1.92	N	312, 217, 186, 173, 144
9	8.69	1-Methyl-2-[(4Z,7Z)-4,7-tridecadienyl]-4(1H)-quinolone	C_23_H_31_NO	338.2484	338.2480	−1.18	N	338, 217, 186, 173,
10	8.95	1-Methyl-2-undecyl-4(1H)-quinolone	C_21_H_31_NO	314.2484	314.2484	0	N	314, 186, 173, 144, 132
11	9.24	Evocarpine	C_23_H_33_NO	340.2640	340.2642	0.59	Y	340, 187, 186, 174, 173, 159, 144, 132
12	9.58	1-Methyl-2-dodecyl-4(1H)-quinolone	C_22_H_33_NO	328.2640	328.2633	−2.13	N	328, 186, 173
13	9.63	1-Methyl-2-[(6Z,9Z)-6,9-pentadecadienyl]-4(1H)-quinolone	C_25_H_35_NO	366.2797	366.2814	4.64	N	354, 328, 305, 261, 217, 186, 173, 144
14	10.25	Dihydroevocarpine	C_23_H_35_NO	342.2797	342.2805	2.34	Y	342, 340, 261, 217, 186, 173, 144, 122
15	10.47	1-Methyl-2-[(Z)-9-pentadecenyl]-4(1H)-quinolone/1-methyl-2-[(Z)-10-pentadecenyl]-4(1H)-quinolone	C_25_H_37_NO	368.2953	368.2942	−2.99	N	368, 342, 217, 186, 173, 144, 122
16	10.54	1-Methyl-2-[(Z)-9-pentadecenyl]-4(1H)-quinolone/1-methyl-2-[(Z)-10-pentadecenyl]-4(1H)-quinolone	C_25_H_37_NO	368.2953	368.2949	−1.09	N	368, 342, 217, 186, 173, 144, 122
17	11.83	1-Methyl-2-pentadecyl-4(1H)-quinolone	C_25_H_39_NO	370.3110	370.3105	−1.35	N	370, 261, 217, 186, 173, 144, 122

(a) Y represents detected in the blood and N denotes not detect.
